# Is the AO guideline for postoperative treatment of tibial plateau fractures still decisive? A survey among orthopaedic surgeons and trauma surgeons in the Netherlands

**DOI:** 10.1007/s00402-017-2718-7

**Published:** 2017-05-22

**Authors:** M. van der Vusse, P. H. S. Kalmet, C. H. G. Bastiaenen, Y. Y. van Horn, P. R. G. Brink, H. A. M. Seelen

**Affiliations:** 1 Adelante Rehabilitation Centre, Hoensbroek, The Netherlands; 2 Department of Traumatology, Maastricht University Medical Centre, P. Debyelaan 25, 6229 HX Maastricht, The Netherlands; 3 Research School CAPHRI, Maastricht University, Maastricht, The Netherlands; 4 Laurentius Hospital Roermond, Roermond, The Netherlands

**Keywords:** Tibial plateau fractures, Trauma patients, Postoperative treatment, Rehabilitation, Weight bearing

## Abstract

**Introduction:**

The standard aftercare treatment (according to the AO guideline) for surgically treated trauma patients with fractures of the tibial plateau is non-weight bearing or partial weight bearing for 10–12 weeks. The purpose of this study was to investigate the current state of practice among orthopaedic surgeons and trauma surgeons in choosing the criteria and the time period of restricted weight bearing after surgically treated tibial plateau fractures.

**Materials and methods:**

A web-based survey was distributed among members of the Dutch Trauma Society and Dutch Orthopaedic Society to identify the most commonly applied protocols in terms of the post-operative initiation and level of weight bearing in patients with tibial plateau fractures.

**Results:**

One hundred and eleven surgeons responded to the survey. 72.1% of the respondents recommended starting weight bearing earlier than the 12 weeks recommended by the AO guideline; 11.7% recommended starting weight bearing immediately, 4.5% after 2 weeks and 55.9% after 6 weeks. Moreover, 88.7% of the respondents reported deviating from their own local protocol. There is little consensus about the definition of 100% weight bearing and how to build up weight bearing over time.

**Conclusion:**

This study demonstrates that consensus about the weight bearing aftercare for tibial plateau fractures are limited. A large majority of surgeons do not follow the AO guideline or their own local protocol. More transparent criteria and predictors are needed to design optimal weight-bearing regimes for the aftercare of tibial plateau fractures.

## Introduction

The incidence of tibial plateau fractures is approximately 13.3 per 100,000 persons [1]. The postoperative management of these surgically treated fractures in trauma patients is of the utmost importance for a full recovery of knee function and the patient’s participation in daily activities and work. Tibial plateau fractures are a cause of long-term disability and pain, and frequently lead to many weeks off work, with substantial economic effects.

The standard aftercare treatment in surgically treated trauma patients with fractures of the tibial plateau is non-weight bearing or partial weight bearing [2]. According to the AO principles of fracture management, postoperative management of tibial plateau fractures generally consists of toe-touch weight bearing for 6–8 weeks. As to fractures caused by extremely high energy; these patients may need to adhere to toe-touch weight bearing regimen for 10–12 weeks [3]. However, there is currently no worldwide consensus among surgeons with regard to permissive weight bearing versus restricted weight bearing in surgical trauma patients with fractures of the tibial plateau [4]. Permissive weight bearing might be early weight bearing, but this is not the goal as such. In permissive weight bearing the patient dictates the progress in weight bearing together with the physiotherapist.

Although biomechanical and animal studies suggest that early weight bearing is beneficial [5–7], there have been virtually no high-quality clinical studies comparing permissive weight bearing (PWB) with restricted weight bearing (RWB) after surgically treated tibial plateau fractures.

The purpose of the present survey was to investigate the current state of postoperative practice among Dutch orthopaedic surgeons and trauma surgeons regarding patients with surgically treated tibial plateau fractures. The survey asked whether they adhered to the AO guideline and their own local guidelines and which criteria they used to decide when and at what level to start weight bearing after surgery.

## Materials and methods

A web-based survey was developed by the authors and was distributed among Dutch orthopaedic surgeons and trauma surgeons, using online software (http://www.formdesk.nl). The survey was publicised at the Dutch trauma congress in 2013 and placed on the websites of the Dutch Trauma Society and the Dutch Orthopaedic Society. Together, the two societies comprise 1293 members. In addition, we approached the surgeons through direct email at their hospital departments in the period of November 2013–October 2014. The survey consisted of twelve questions, shown in Table 1.

**Table 1:** The questionnaire

1. What is your discipline?
2. How long have you been a surgeon?
3. How often do you operate a tibial plateau fracture on yearly basis?
4. When do you start aftercare weight bearing in patients with tibial plateau fractures and with which weight bearing percentage?
5. Do you occasionally deviate from the standard postoperative protocol used in your clinic?
6. If you deviate from the standard protocol, on which factors is your decision based?
7. Which criteria do you use to determine earlier or delayed weight bearing?
8. How do you define 100% weight bearing?
9. How do you (gradually) increase postoperative weight bearing?
10. What kind of early complications do you see in patients with tibial plateau fractures in your clinic?
11. Are these complications related to early weight bearing?
12. Do you see yourself as a surgeon who is a more conservative or more progressive in the aftercare of tibial plateau fractures?

### Statistical analysis

Statistical analysis was performed using IBM SPSS Statistics, Version 23.0, Armonk, NY. Descriptive statistics were used to describe the demographic data and baseline characteristics of the entire survey. Results are presented as either mean ± standard deviation (SD) or as frequencies and percentages.

## Results

Of the 111 surgeons who responded in the survey, 61 (55.0%) were orthopaedic surgeons and 50 (45.0%) were trauma surgeons. The overall response rate was 8.6% (i.e. 111/1293). Thirty-eight (34.2%) respondents were active as a surgeon for 0–5 years, *N* = 21 (19.0%) for 5–15 years, and *N* = 52 (46.8%) for more than 15 years. Forty-four surgeons (39.6%) operated less than 5 times per year on patients with a tibial plateau fracture, 51 surgeons (46.0%) operated between 5 and 10 times per annum on these patients, and 16 (14.4%) more than 10 times per year on these patients.

**Fig. 1:**
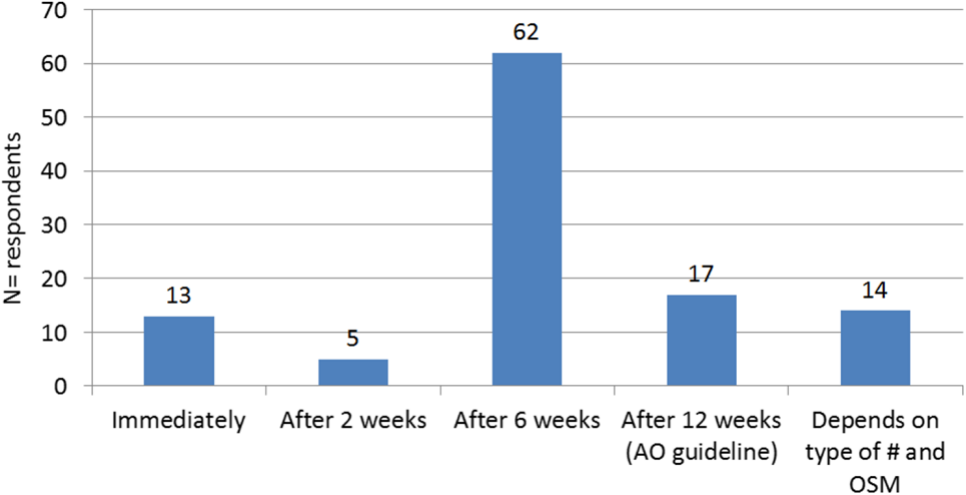
When do you start weight bearing after tibial plateau fractures and with what weight bearing percentage? # fracture, *OSM* osteosynthesis material

Surgeons were asked when they started weight bearing after surgical treatment of tibial plateau fractures and with which weight bearing percentage. The results are shown in Fig. 1: 11.7% of the respondents started immediately with weight bearing, 4.5% after 2 weeks, while the majority (55.9%) recommended starting weight bearing 6 weeks post-operatively. Only 15.3% recommended weight bearing after 12 weeks, i.e. in line with the AO guideline. Furthermore, 12.6% of the respondents recommended that the start of weight bearing should depend on the type of fracture and the osteosynthesis material. These findings imply that 72.1% of the respondents recommended starting weight bearing earlier than the 12-week period recommended by the AO guideline.

**Fig. 2:**
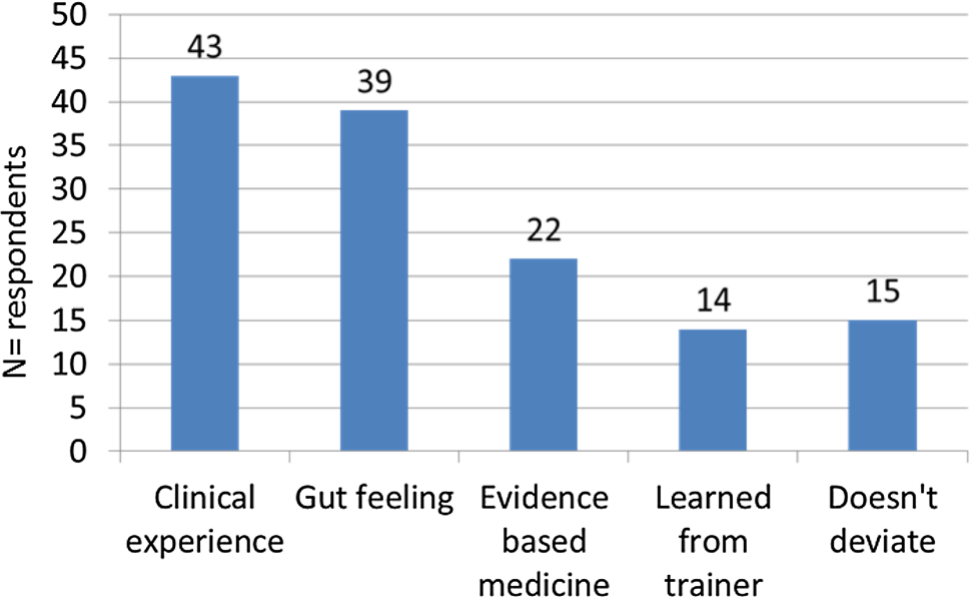
Reasons for deviating from own standard local protocol

Figure 2 show that 88.7% of the respondents occasionally deviated from their local standard protocol, in most cases based on clinical experience (38.7%) and gut feeling (35.1%), while 19.8% of the respondents deviated on the basis of the so-called evidence-based medicine, even though the latter is scarce in the literature.

**Fig. 3:**
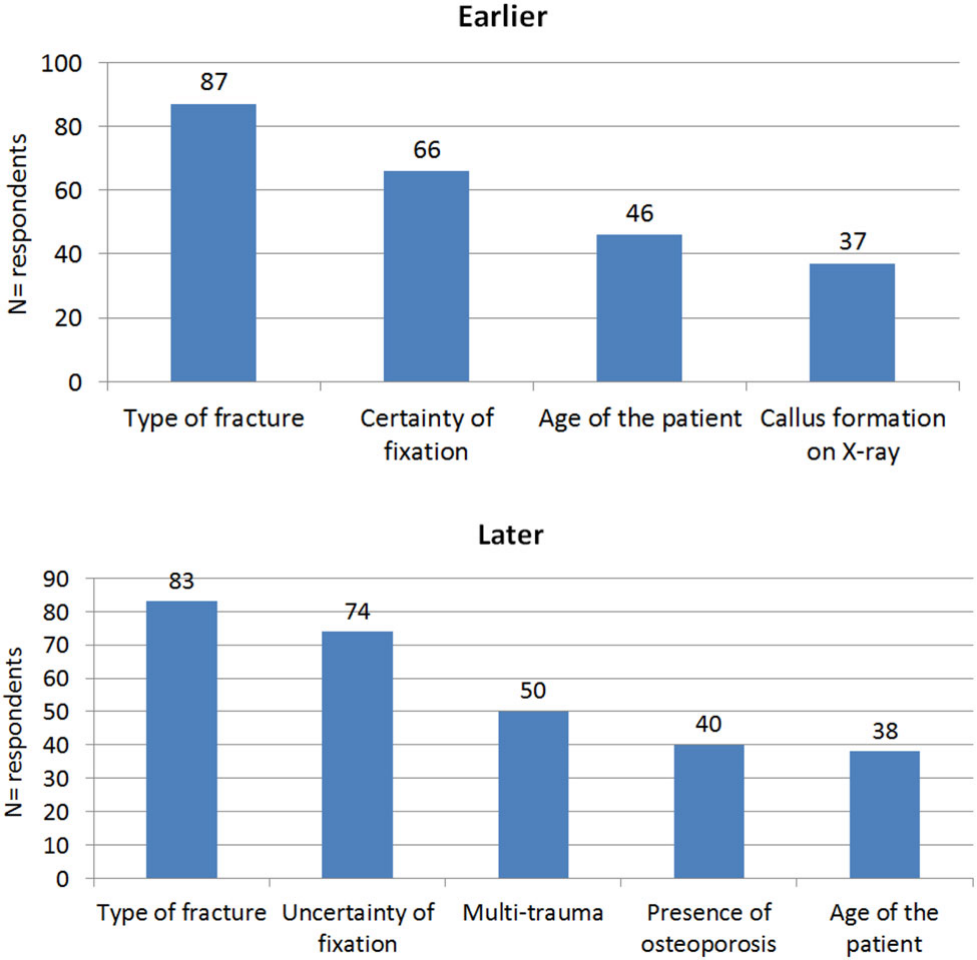
Which criteria are used to decide earlier or later weight bearing?

Frequently mentioned reasons for starting weight bearing earlier or later were fracture type [*N* = 87 (78.4%) and *N* = 83(74.8%), respectively], certainty or uncertainty of fixation quality [*N* = 66 (59.5%) and *N* = 74 (66.7%), respectively], age [*N* = 46 (41.4%) and *N* = 38(34.2%), respectively] and additional traumata [*N* = 0 (0.0%) and *N* = 50(45%), respectively]. The type of fracture and the certainty or uncertainty of the fixation are the two most important aspects that influence the prescription of early (or later) weight bearing regimen (Fig. 3).

**Fig. 4:**
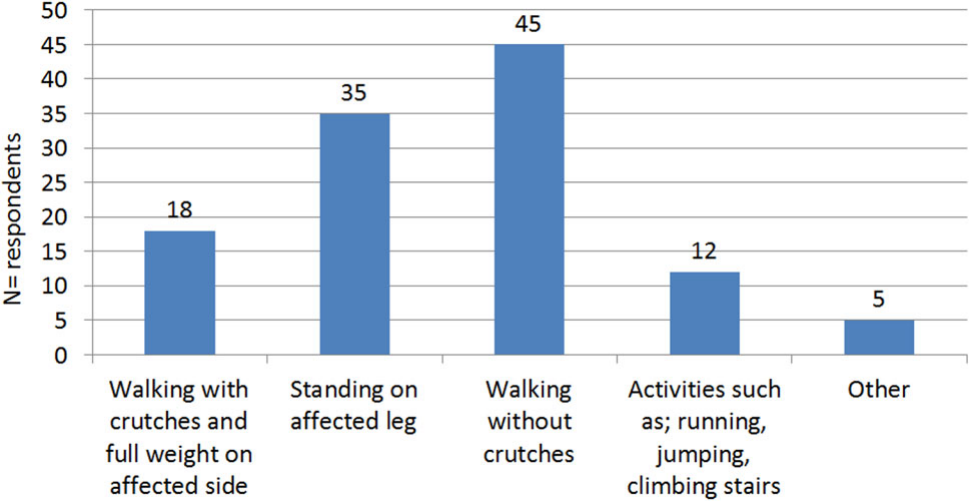
Definition of “100% weight-bearing” used by surgeons

Surgeons who recommended starting weight bearing immediately or after 2 weeks mostly commenced with a low dosage (10–25%) of weight bearing (Table 2). If weight bearing started 6 weeks post-operatively, this was mainly at 50% (27 respondents) or 10–25% (21 respondents) of the maximum level. When patients started weight bearing 12 weeks after the surgical treatment, 10 out of 11 surgeons recommended starting immediately with 100% weight bearing. Since it is important to know what surgeons regard as “100% weight bearing”, we asked for their definition of “100% weight bearing”, results of which are shown in Fig. 4. The majority, i.e. 45 (40.5%) respondents, defined this as “walking without crutches”, 35 (31.5%) respondents indicated “standing on one leg of the affected side”, 20 (18.0%) respondents mentioned “walking with crutches” and 10 (9.0%) respondents considered “100% weight bearing” to be “running, jumping, climbing a staircase”.

**Table 2:** Level of weight bearing (percentage) patients are allowed to start with

Maximal weight bearing (%)	Direct/early weight bearing	After 2 weeks	After 6 weeks	After 12 weeks (AO-guideline)	Depends on type # and OSM
10–25%	10	4	21	0	0
50%	1	0	27	1	0
75%	0	0	1	0	0
100%	1	1	6	10	0
Weight bearing without%	1	0	7	6	14
Total	13	5	62	17	14

# fracture, *OSM* osteosynthesis material

**Fig. 5:**
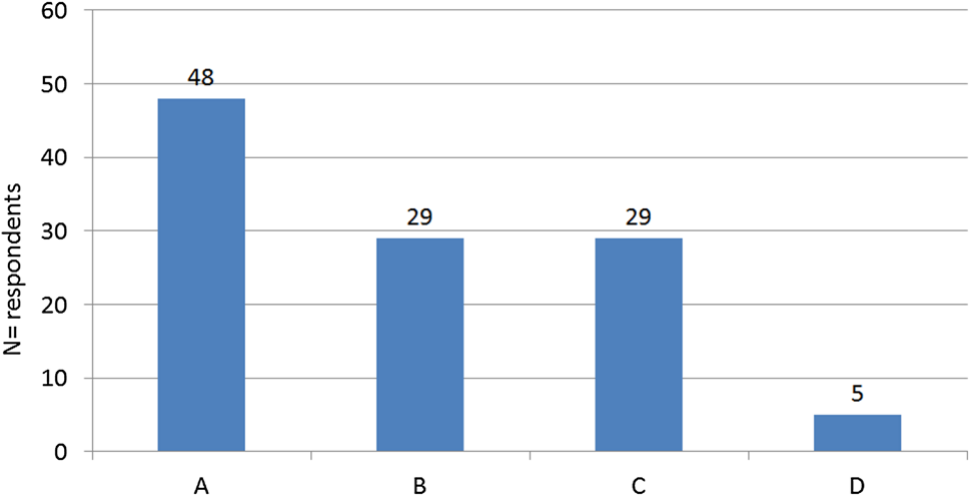
How do surgeons advise their patients about increasing weight bearing following a graded protocol? *A* Graded increase over time in a fixed number of weeks, expressed in kilogram or percentage of body weight. *B* Graded increase over a fixed number of weeks, expressed as much weight bearing as tolerated by the patient. *C* Permissive weight bearing, which means surgeons let patients and therapists decide how to build up the weight bearing as tolerated. *D* Other. *RROM* restricted range of motion

Most surgeons (*N* = 48, 43.2%) told their patients that weight bearing should increase gradually over a fixed number of weeks, expressed in kilograms or percentage of body weight. Twenty-nine (26.0%) surgeons recommended gradually increasing weight over a fixed number of weeks to a level of 100%, based on how much weight bearing the patient could tolerate. Twenty-nine (26.0%) surgeons recommended permissive weight bearing, which means surgeons let patients and therapists decide how to build up the weight bearing as tolerated (Fig. 5). Of the 29 (26.0%) respondents who recommended permissive weight bearing, *N* = 12 (10.8%) were orthopaedic surgeons and *N* = 17 (15.3%) were trauma surgeons. Eight (7.2%) respondents who recommended permissive weight bearing had a work experience of 0–5 years, *N* = 14 (12.6%) 5–15 years and *N* = 7 (6.3%) more than 15 years. In this group 9 surgeons (8.1%) operated on these patients less than 5 times per year, 16 (14.4%) between 5 and 10 times per year, and 4 (3.6%) more than 10 times per year. Fifty-three (47.7%) respondents were conservative in the aftercare of tibial plateau fractures and *N* = 58 (52.3%) were progressive in the aftercare.

## Discussion

This survey is an attempt to obtain up-to-date information on the time period between surgical treatment of tibial plateau fractures and the start of rehabilitation involving weight bearing. The AO guideline for postoperative management of tibial plateau fractures was formulated about 50 years ago and suggests restricted weight bearing for approximately 12 weeks [3]. It is generally assumed that orthopaedic surgeons and trauma surgeons follow the AO guideline, advocating restricted weight bearing during aftercare for the patients. Interestingly, the present study shows that a large proportion of orthopaedic surgeons and trauma surgeons in the Netherlands recommended starting weight bearing earlier than 12 weeks. In practice, the vast majority of the responding surgeons deviated from their own institutional guidelines, based on clinical experience and gut feeling, thus deviating from the AO guideline.

The period of delayed weight bearing was reported to depend, inter alia, on the fracture type, certainty or uncertainty about fixation quality and additional traumata. To date, we have not been able to identify studies providing methodologically sound evidence as to critical factors that may assist in the decision to start weight bearing earlier or later. However, many studies have shown a trend towards favouring earlier weight bearing. Long-term outcomes have also been described in the literature, with no negative effects of early weight bearing being reported [8–13].

It is important to note that this study did not intend to determine the optimal aftercare for a given tibial plateau fracture, but was designed to disclose the current practice regarding tibial plateau fracture surgery aftercare and the factors on which orthopaedic surgeons and trauma surgeons found their decisions.

This study clearly demonstrates that there is as yet no consensus about the aftercare of tibial plateau fractures. Furthermore, there is no evidence to restrict patients in bearing weight for 10–12 weeks as suggested by the AO guideline. Our findings show that at least in the Netherlands, the AO guideline is not decisive. In addition, we found large variations in post-operative rehabilitation treatment.

It should be kept in mind that another complicating factor could be lack of patient compliance with prescribed rehabilitation aftercare [14, 15]. A number of studies reported that patients often exceeded the prescribed level of partial weight bearing, even when self-reported compliance was high [16]. Thus, despite the expressed willingness to comply, patients often do not follow the restrictions on weight bearing and increase their weight bearing as fracture healing progresses. Together with the finding that there is no consensus as to what the definition of “100% weight bearing” is and how to build up weight in a protocolled way, our study revealed a large diversity in practical weight bearing usage among surgeons. This makes it even more difficult to achieve a good interpretation of the aftercare and offer customized advice to patients regarding the optimal aftercare in terms of weight bearing during the rehabilitation.

There are a few important limitations to this study. The study is limited regarding the response rate. The survey did not describe the different types of fractures and assumptions regarding the energy of trauma. Furthermore, it is important to note that this study does not attempt to describe what the correct aftercare treatment is in tibial plateau fractures. It rather focuses on obtaining up-to-date information on the time period between surgical treatment of tibial plateau fractures and the start of rehabilitation involving weight bearing. In summary, the outcome of this survey shows that there is no clear consensus about optimal postoperative treatment of patients with a tibial plateau fracture, which may result in suboptimal rehabilitation aftercare. This leaves open the question what is the optimal rehabilitation treatment in surgically treated tibial plateau fractures. To answer this question, the authors recommend that both the AO guideline and current local institutional guidelines should be critically scrutinized to establish the optimal aftercare for these patients. In theory, it is normally the surgeon who decides which aftercare protocol should be followed. Most often, this is a restricted weight bearing regime with a build up time over a fixed number of weeks. In practice, such protocols are not followed very strictly. In our study we found that 26% of the respondents would like to advocate using a customized permissive weight bearing protocol, which is in line with studies by Solomon et al. and Segal et al. who also support individualized permissive weight bearing [13, 17]. Furthermore, no clear differences as to adherence to the permissive weight bearing were found between either orthopaedic & trauma surgeons, and between groups of surgeons with different levels of work experience. However, high-quality prospective studies are needed to help identify which criteria and predictive factors are important for developing a (permissive) weight bearing protocol to optimize patients’ comfort and optimize the course of recuperation.

## Conclusion

This study demonstrates that consensus about the weight bearing aftercare for tibial plateau fractures are limited. A large majority of surgeons do not follow the AO guideline or their own local protocol. More transparent criteria and predictors are needed to design optimal weight-bearing regimes for the aftercare of tibial plateau fractures.
